# A novel parvovirus circulating in canine populations and sporadically detected in human oropharyngeal samples

**DOI:** 10.1128/spectrum.03327-25

**Published:** 2026-02-09

**Authors:** Xiang Lu, Ning Kong, Chunmei Wang, Juan Lu, Wang Li, Hongfeng Yang, Xiaoxiao Lu, Zheyuan Zhang, Yue Chen, Shiyin Huang, Chenglin Zhou, Yu Zhang, Wen Zhang, Tongling Shan

**Affiliations:** 1 Institute of Critical Care Medicine, The Affiliated People’s Hospital, Jiangsu University 196541 , Zhenjiang, China; 2 Shanghai Veterinary Research Institute, Chinese Academy of Agricultural Sciences 118161 , Shanghai, China; 3 Department of Preventive Dentistry, Shanghai Ninth People’s Hospital, Shanghai Jiao Tong University School of Medicine 56695, Shanghai, China; 4 Clinical Laboratory Center, The Affiliated Taizhou People's Hospital of Nanjing Medical University 372209 , Taizhou, China; 5 Department of Microbiology, School of Medicine, Jiangsu University 597611, Zhenjiang, China; University of Prince Edward Island, Charlottestown, Prince Edward Island, Canada

**Keywords:** parvovirus, canine, human, recombination

## Abstract

Most human pathogens, while originating from animals, have crossed species barriers to infect humans, often leading to outbreaks of new infectious diseases. Despite significant efforts, the mechanisms, timing, and locations of these emerging diseases remain largely uncertain. Here, using a viral metagenomic approach, we discovered a novel canine-associated parvovirus in human oropharyngeal secretions. Molecular screening revealed the presence of this parvovirus in different canine tissues, including 24 of 108 pharyngeal lymph node samples. Further molecular investigation showed that the virus was detected in the oropharyngeal secretions of pet dogs and in human samples that were not linked to these animals. This parvovirus was therefore named human-canine associated parvovirus 1 (HCAPV-1). Nine complete genomes of HCAPV-1 were acquired through next-generation sequencing, combining Sanger sequencing. Genomic and phylogenetic analyses indicate that these nine strains of HCAPV-1 belong to the genus *Protoparvovirus* and form a distinct clade, with their closest relatives being newlaviruses from foxes. Amino acid substitutions have been characterized in the capsid proteins of the variants of HCAPV-1, which potentially alter their infection patterns. Potential genomic recombination was also observed in HCAPV-1. Taken together, our findings reveal the presence of a novel parvovirus in both canine and human samples, highlighting the need to investigate its host range and transmission dynamics.

**IMPORTANCE:**

This study identified a novel parvovirus, human-canine associated parvovirus 1 (HCAPV-1), which was detected in human oropharyngeal secretions and various canine tissues, suggesting that its host range may extend beyond a single species. Phylogenetic analysis revealed that HCAPV-1 forms a distinct clade within the genus *Protoparvovirus*, closely related to newlaviruses from foxes. Amino acid substitutions observed in the capsid proteins of HCAPV-1 variants indicate genetic divergence, warranting further investigation into their potential implications for host interactions. Recombination events may have contributed to its emergence. This finding highlights the importance of continued surveillance in settings where humans and companion animals coexist and underscores the need for further research to clarify the ecological and host-range characteristics of such viruses.

## INTRODUCTION

Members of the family *Parvoviridae*, which currently includes at least 3 subfamilies, 28 genera, and 188 species (https://ictv.global/taxonomy), have been detected in nearly all major vertebrate clades, as well as in both protostome and deuterostome invertebrates (1). Among those receiving widespread attention are viruses within the subfamily *Parvovirinae*, which infect vertebrates, including mammals (2), birds (3), and reptiles (4). The first parvovirus identified in humans was the adeno-associated virus (5). Since then, several other parvoviruses have been discovered in human samples, including parvovirus B19, bocavirus 1-4, parvovirus 4, bufavirus, tusavirus, and cutavirus (6, 7). While some human parvoviruses are known to cause disease during acute infections, others have been associated with chronic conditions (7, 8).

Recent studies have significantly expanded our understanding of the diversity of parvoviruses and highlighted their evolving ability to adapt to a broader range of hosts (9). Moreover, cross-species transmission of parvoviruses among animals is common. For example, Zhao et al. identified a novel parvovirus related to goose parvovirus in diseased ostriches, with this host jump likely driven by recombination and nucleotide deletions in the inverted terminal repeat region (10). Additionally, it has been reported that *Carnivore Protoparvovirus 1* (CPPV-1), which includes the well-known canine parvovirus (CPV) and feline panleukopenia virus (FPV) that were first transmitted among human companion animals, such as canines and felines (11), has now crossed over to giant pandas (12), pigs (13), lions (14), pangolins (15), and raccoons (16). So far, direct instances of parvovirus spilling over from animal hosts to humans have not been observed. Few reports exist, such as Reuter et al.’s identification of tusavirus in the feces of domestic goats and sheep, a virus closely related to the human stool-associated strain (17).

## RESULTS

### Finding a novel parvovirus in human oropharyngeal secretions

To investigate the diversity of viruses in the human oropharynx and evaluate their potential health impacts, we initiated the “Metaviromic Project of Human Oropharyngeal Secretions.” As part of this project, we performed next-generation sequencing (NGS) on more than 200 human oropharyngeal secretion samples collected between 2020 and 2022. Surprisingly, we identified an almost complete parvovirus genome in one of the libraries (Library ID HOS114; CRA accession no. CRA018894), with BLASTn analysis revealing its closest match to be the Newlavirus strain ITA/2013/51.20-65 (GenBank accession no. ON959793), a member of the genus *Protoparvovirus* that was detected in the retropharyngeal lymph node of a fox in Italy (18), sharing 75.59% nucleotide sequence identity.

Since detecting a novel eukaryotic virus with such low identity in human oropharyngeal secretions is uncommon, and considering that the oropharynx is a critical site for the infection and replication of most respiratory viruses (19), we used the genome of this virus as a reference to map against all other libraries to determine whether its presence was coincidental. As a result, sequence reads of this novel parvovirus also exist in the other two libraries, i.e., HOS115 and HOS116, with 13 and 53 mapped sequence reads, respectively. Through Sanger sequencing, we confirmed the complete genome of this parvovirus, which is 4,989 bp in length.

### Tracing this novel parvovirus in canine populations

Given the close genetic relationship between this parvovirus and newlaviruses found in foxes, which have been widely detected in fox lymph nodes (18), we therefore conducted a molecular tracing study of this virus in canine populations, as both dogs and foxes belong to the family *Canidae*. We first tested 108 previously preserved canine lymph node samples and whole-body tissue samples from three deceased stray dogs (see Materials and Methods for details). PCR screening for a 244-nt VP gene fragment, which is essential for infectivity (20, 21), revealed that 24 out of 108 canine lymph node samples were positive, resulting in a positivity rate of 22.22%. In one of the deceased stray dogs, the virus was detected in multiple tissues, including the submandibular lymph node, retropharyngeal lymph node, brain, spleen, mesenteric lymph node, inguinal lymph node, stomach, small intestine, and blood. This finding suggests that the novel parvovirus has extensive infection characteristics and a potential for systemic spread. Finding this parvovirus in both human and dog samples, we then named it human-canine associated parvovirus 1 (HCAPV-1).

### Dog-derived HCAPV-1 genomes acquisition

To get complete genomes of HCAPV-1 from dog samples, we performed NGS for the HCAPV-1-positive canine lymph node samples. We then mapped the complete HCAPV-1 genome to the NGS data to acquire potential homologous genomes. However, most libraries only contain a small number of sequence reads mapping to the genome of HCAPV-1, reflecting the low viral load of HCAPV-1 in the dog lymph node samples. Subsequently, using the human-derived HCAPV-1 genome as a template, we designed primers and performed whole-genome amplification (Table S1), followed by Sanger sequencing. This approach yielded eight parvovirus sequences homologous to HCAPV-1 from canine lymph node tissues. The lengths of these sequences range from 4,795 to 4,807 bp, with pairwise nucleotide identities exceeding 98%, and all the dog-derived HCAPV-1 genomes share over 96% nucleotide sequence identity with the complete HCAPV-1 genome (Fig. S1). Overall, we acquired nine different genomic sequences of HCAPV-1.

### Genome characterization of HCAPV-1

**Fig 1:**
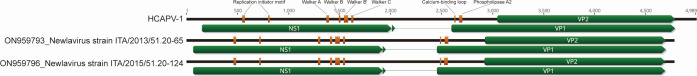
Comparative analysis of the genomic organization of HCAPV-1 and related newlaviruses. The gene organization of HCAPV-1, Newlavirus strain ITA/2013/51.20-65, and Newlavirus strain ITA/2015/51.20-124 is depicted. Predicted conserved motifs are highlighted in yellow boxes.

The genome organization of HCAPV-1 was determined through BLASTn alignment with its closest relatives, two newlaviruses (genus *Protoparvovirus*) associated with foxes (GenBank accession numbers ON959793 and ON959796). Based on the HCAPV-1 genome from human oropharyngeal secretion (GenBase no. C_AA085195.1), this virus exhibits a typical protoparvovirus organizational pattern (Fig. 1), with a GC content of 40.0% and a nucleotide composition consisting of 36.3% A, 23.6% T, 18.5% G, and 21.6% C. Specifically, HCAPV-1 possesses a partial 5′ untranslated region (150 bp), a complete open reading frame (ORF) for non-structural protein 1 (NS1) consisting of 620 amino acids (aa), complete ORFs for virus proteins VP1 (740 aa) and VP2 (581 aa), and a partial 3′ untranslated region (201 bp). The HCAPV-1 genome contains highly conserved protein domains, including motifs in the NS1 protein that are related to ATP- or GTP-binding. These motifs include the Walker A loop (GxxxxGKT/S; GPASTGKS), the Walker B motif (uuuuEE; IIWVEE), the Walker B′ motif (KxxxxGxxxxxxxK; KAICSGQSIRIDQK), and the Walker C motif (PxxxTxN; PVIITTN) (22) (where x represents any aa, and u represents an uncharged aa) (3, 23). In addition, the NS1 protein contains two conserved replication initiator motifs: xxHuHxxxx (KLHIHVLLH) and YxxxK (YFLQK). The phospholipase A2 (PLA2) catalytic residues DxxAxxHDxxY (DAAAQRHDHAY) and its highly conserved calcium-binding loop (GPGN) were found at the N-terminus of the VP1 (17).

### Further investigation of HCAPV-1 in human and canine oropharyngeal secretions

To further examine the presence of HCAPV-1 in humans and dogs that had close contact, we collected 126 oropharyngeal secretion samples from pet dogs and 62 additional samples from their close human contacts for viral detection using the specific PCR primers mentioned above. Additionally, we also tested 270 oropharyngeal secretion samples from febrile individuals to investigate any association between HCAPV-1 infection and fever. The results indicated that 3 out of 126 oropharyngeal samples from pet dogs tested positive, with a positivity rate of 2.38%. Among 62 human samples collected from individuals who had close contact with pet dogs, 2 tested positive (3.23%). Notably, although the sampling framework included both pet dogs and their close human contacts, the human and canine positive cases were not from corresponding human-dog pairs. In addition, all oropharyngeal samples from febrile patients tested negative (Table 1).

**TABLE 1:** Screening for HCAPV-1 positivity in human and canine populations

Sample type	Total samples	Positive samples	Positivity rate (%)
Canine lymph nodes	108	24	22.22
Deceased stray dog whole-body tissues	3	Multiple tissues from a single dog	N/A
Oropharyngeal secretions from pet dogs	126	3	2.38
Oropharyngeal secretions from close contacts of pet dogs	62	2	3.23
Oropharyngeal secretions from febrile patients	270	0	0

### Phylogenetic analysis of HCAPV-1 and its diverse variants

**Fig 2:**
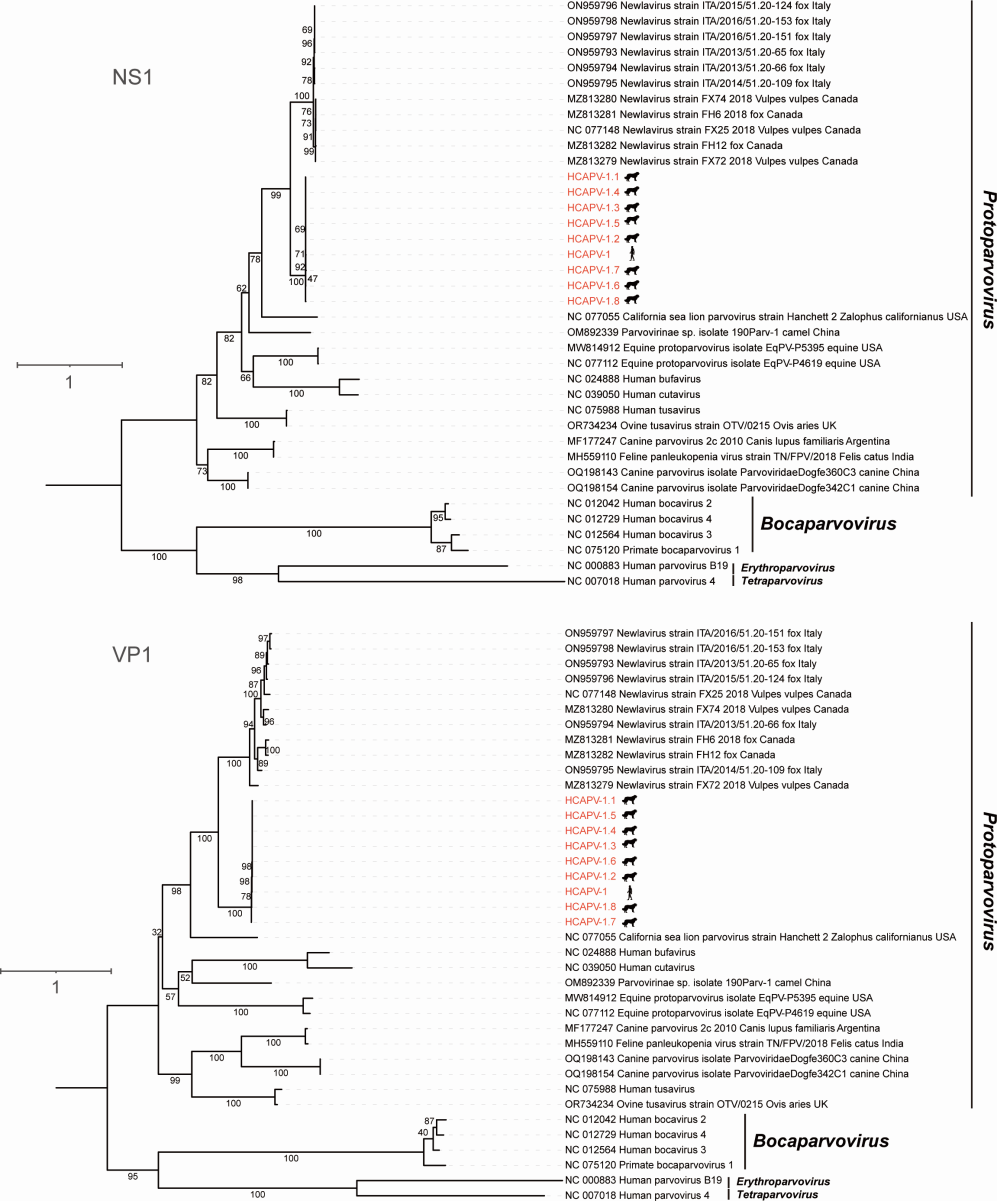
Phylogenetic analysis of HCAPV-1 and its diverse variants. The maximum likelihood phylogenetic trees were generated based on the NS1 and VP1 protein sequences of parvoviruses. Viruses identified in this study are highlighted in red. Numbers along the branches indicate the percentage of bootstrap values. The scale bar represents the number of substitutions per site. All animal and other life form silhouettes are sourced from PhyloPic (https://www.phylopic.org) and are available for reuse under Creative Commons licenses.

To determine the evolutionary relationships between HCAPV-1, its variants, and previously identified parvoviruses, we estimated phylogenetic trees based on nucleotide sequences of the whole genomes, as well as the NS1 and VP1 genes, and the proteins encoded by these genes (Fig. 2; Fig. S2). In all phylogenetic analyses, HCAPV-1 and its variants consistently exhibited a close relationship with members of the Newlavirus group and clustered robustly within the genus *Protoparvovirus*. Distance matrix analysis of the NS1 protein, performed using SDT v1.3 (24), revealed that HCAPV-1 and its variants share no more than 77% aa identity with newlaviruses (Fig. S3). According to the ICTV criteria for species classification within the genus *Protoparvovirus*, viruses of the same species must be monophyletic and encode an NS1 protein with more than 85% aa sequence identity (https://ictv.global/report/chapter/parvoviridae/parvoviridae/protoparvovirus). Therefore, HCAPV-1 and its variants should be classified as a novel species.

### Secondary structure prediction

**Fig 3:**
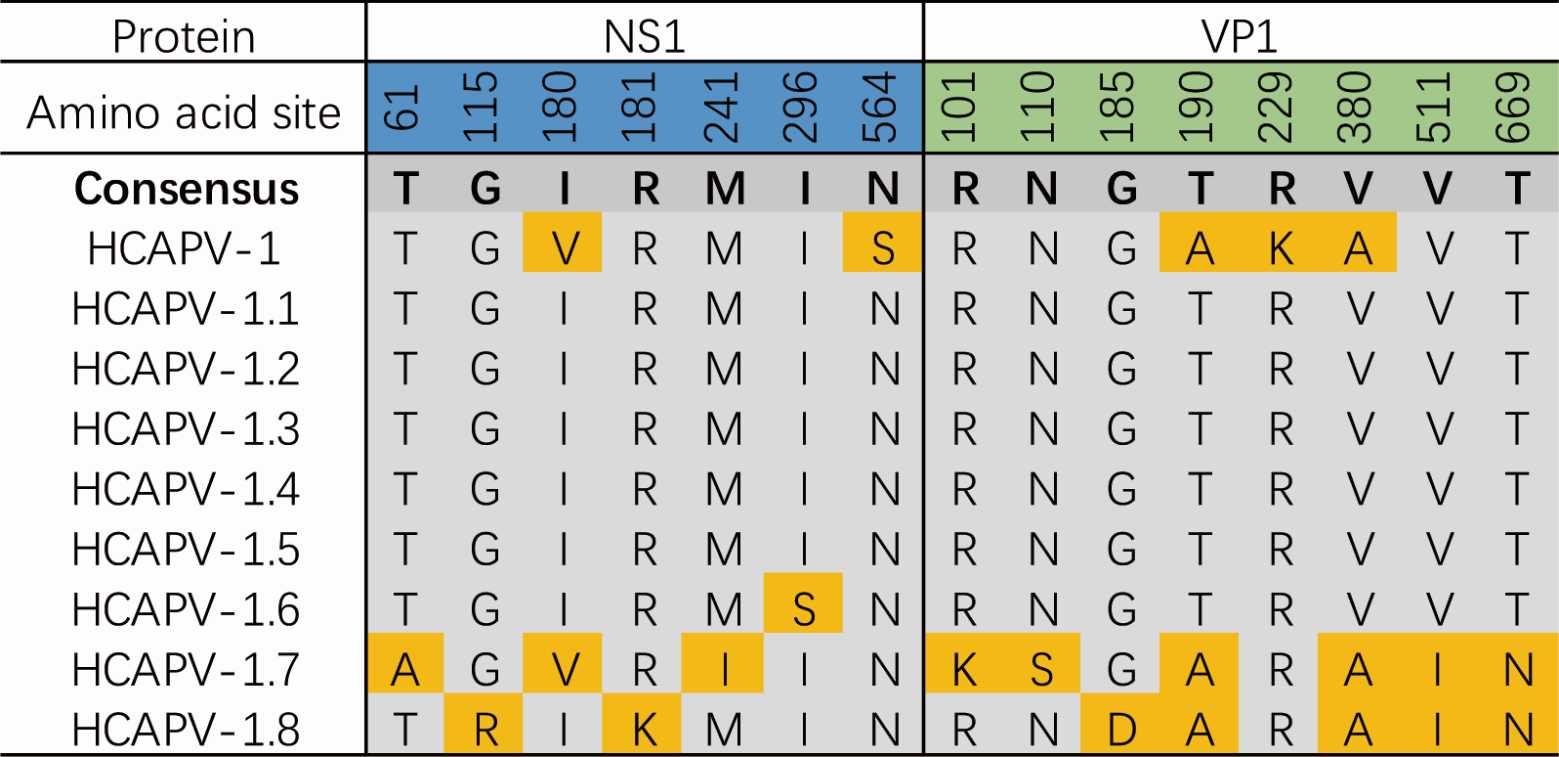
The amino acid substitution sites in the NS1 and VP1 proteins of HCAPV-1 and its variants. Substitution sites relative to the consensus sequence are highlighted in yellow, while conserved sites are shaded in gray.

Many viral sequences exhibit host-specific mutations in their capsid proteins and demonstrate frequent parallel evolution (25). Changes in the secondary structure of proteins, such as α-helices and β-sheets, may lead to alterations in the three-dimensional conformation, potentially resulting in the loss or modification of protein function. A classic example of this is the interaction between the transferrin receptor (TfR) and CPV/FPV, as both viruses use TfR to bind to and infect cells (20). FPV binds exclusively to feline TfR, whereas CPV is capable of binding to both canine and feline TfR. The DNA sequence divergence between FPV and CPV isolates is as low as 0.5%, with critical differences, including a substitution of Lys to Asn at VP2 residue 93 and Asp to Asn at VP2 residue 323. These specific mutations are sufficient to confer upon CPV the ability to infect the canine host (26). In order to explore potential differences in the spatial structures of HCAPV-1 and its variants, we used the Garnier program within the EMBOSS (27) suite to predict the secondary structures of their NS1 and VP1 aa sequences (Fig. S4). The results revealed that the nine viruses had seven aa differences in NS1 and eight in VP1, likely due to VP1 being relatively less conserved. These aa variations resulted in alterations in the secondary structure of at least four regions in NS1 and VP1, potentially affecting protein function. For zoonotic viruses, functional changes in the VP proteins may pose significant public health risks. Notably, compared to the consensus sequence, HCAPV-1.7 exhibited nine aa substitutions (three in NS1 and six in VP1), indicating a more complex pattern of variation relative to other homologous sequences. Following this, HCAPV-1.8 had seven aa substitutions (two in NS1 and five in VP1), HCAPV-1 had five substitutions (two in NS1 and three in VP1), and HCAPV-1.6 had one substitution (in NS1) (Fig. 3).

### Genomic recombination analysis of HCAPV-1

**Fig 4:**
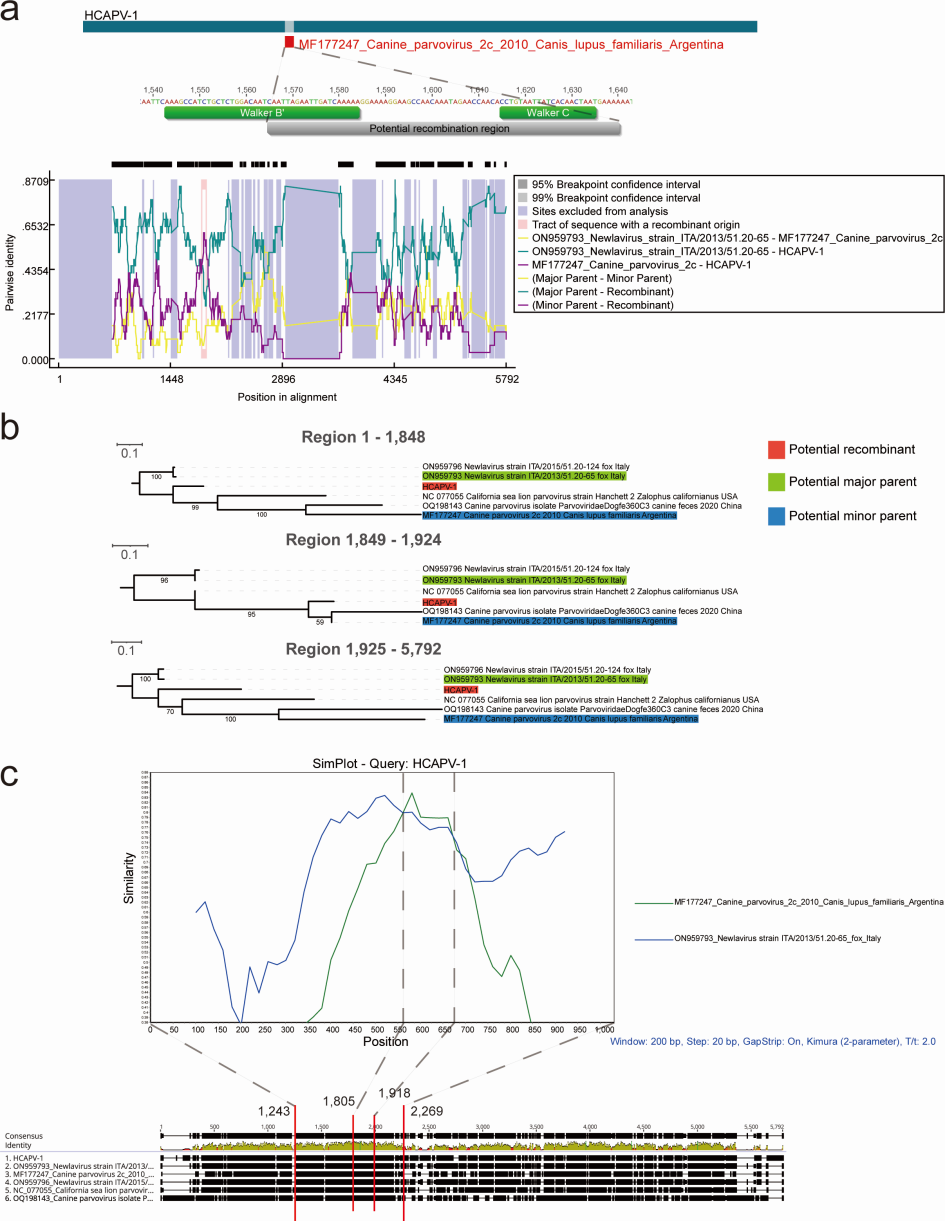
Putative recombination events identified in the NS1 gene of HCAPV-1. (**a**) Recombination events in the HCAPV-1 genome were predicted using RDP5. The genome is depicted as a dark blue bar, with regions involved in recombination events highlighted in red. Predicted recombination breakpoints are clearly indicated along the genome. (**b**) The phylogenies of the major parental regions (positions 1–1,848 and 1,925–5,792) and the minor parental region (positions 1,849–1,924) were inferred using the maximum likelihood method. Numbers along the branches represent bootstrap support values (as percentages). The scale bar indicates the number of substitutions per site. (**c**) The sequence similarity plot of HCAPV-1, Canine parvovirus 2c, and Newlavirus strain ITA/2013/51.20-65 reveals putative recombination events. HCAPV-1 served as the query sequence.

Genomic and phylogenetic analyses suggest that HCAPV-1 might result from cross-infection or share a common virus source between foxes and other canids. To further characterize the putative recombination events in the evolution of protoparvoviruses, the whole genomic sequences of HCAPV-1 and five other viruses (including Canine parvovirus 2c, Canine parvovirus isolate ParvoviridaeDogfe360C3, California sea lion parvovirus strain Hanchett_2, and two Newlavirus strains: ITA/2013/51.20-65 and ITA/2015/51.20-124) were analyzed using the Recombination Detection Program v5.58 (RDP5) (28). These sequences were selected based on BLASTn results and phylogenetic analysis. Only potential recombination events detected by two or more algorithms and supported by phylogenetic evidence of recombination were considered significant, using a *P* value cutoff of 0.05 as the highest acceptable threshold (29). The results indicated that three out of nine detection methods supported recombination in the region between positions 1,849 and 1,924 in the alignment, corresponding to a 76 bp fragment (positions 1,565 to 1,640) in the HCAPV-1 genome, with *P* values ranging from 1.309 × 10^−13^ to 2.836 × 10^−2^ (Fig. 4a; Table S2). Notably, the nucleotide sequence encoding the partial Walker B′ motif and the complete Walker C motif in the HCAPV-1 genome is located within this putative recombination region, potentially influencing the ATP binding, hydrolysis, and DNA translocation activities of the associated proteins (30). In the phylogenetic analysis, HCAPV-1 is most closely related to newlaviruses in the nucleotide fragments from 1 to 1,848 and from 1,925 to the end of the sequence. However, between nucleotides 1,849 and 1,924, HCAPV-1 shows the closest relationship to Canine parvovirus 2c, identified in 2010 in *Canis lupus familiaris* from Argentina (Fig. 4b). Additionally, similarity plot analysis using SimPlot software was performed on the potential recombination regions to further confirm the presence of recombination events within the HCAPV-1 genome (Fig. 4c). Overall, although it cannot be definitively concluded that recombination facilitated the emergence of HCAPV-1, these results at least suggest that Newlavirus and Canine parvovirus 2c were involved in the process. Interestingly, to the best of our knowledge, Newlaviruses have only been detected in foxes from Italy (18) and Canada (31) so far, which are presumed to be their natural hosts. Moreover, considering the significant time gap between the initial identification of these two viruses and their distribution across different continents, it is possible that some related sequences remain undiscovered. Further sampling and identification efforts are needed to better understand their evolutionary relationship with HCAPV-1.

## DISCUSSION

A virus’s host range, which refers to the variety of species it can infect, is critical for understanding its potential to drive emerging diseases. However, defining host range is challenging, as it depends on factors such as host susceptibility and the virus’s ability to sustain transmission in new hosts (25). In this study, HCAPV-1 sequences detected in humans showed high genetic similarity to those identified in dogs, suggesting that the virus may circulate across both host groups. However, this observation does not necessarily imply direct transmission between dogs and humans. Alternative explanations should also be considered, such as exposure of both species to a shared environmental source, including contaminated surfaces or fomites. Given the low detection rate and the limited metadata available for the samples, our ability to distinguish among these possibilities is restricted. Additional epidemiological data, particularly temporally and spatially linked sampling and broader environmental surveillance, will be required to clarify these patterns.

Phylogenetic analyses showed that HCAPV-1 and its variants diverge markedly from the recently reported newlaviruses in Canada and Italy, underscoring how little is known about the evolutionary history and transmission ecology of these viruses. In addition, the VP gene of HCAPV-1 displayed remarkable genetic diversity, which contrasts with the strong conservation typically observed in other protoparvoviruses, such as CPPV-1, where even a few point mutations can lead to significant biological effects (32, 33). These findings highlight the complexity of HCAPV-1 evolution and emphasize the need for further investigation to clarify the functional importance of this variability.

Larger data sets with broader geographic coverage, more extensive sampling, and long-term observations will be important for clarifying the distribution and host range of HCAPV-1 and for assessing any possible clinical relevance. As additional data accumulate, our understanding of parvovirus diversity and evolution is likely to become more comprehensive. The detection of HCAPV-1 in both humans and dogs also highlights the need for continued surveillance in environments where people and companion animals interact closely.

## MATERIALS AND METHODS

### Collection of samples and library construction

HCAPV-1 was initially identified from a subset of data generated by the “Metaviromic Project of Human Oropharyngeal Secretions,” which has not yet been published. Briefly, human oropharyngeal secretions were collected using disposable cotton swabs, stored in sterile containers, and immediately transported to the laboratory on dry ice.

Before conducting metaviromic analysis, the tips of the collected swabs were immersed in 0.5 mL of Dulbecco’s phosphate-buffered saline, vortexed vigorously for 5 min, and then incubated at 4°C for 30 min. After centrifugation at 15,000 × *g* for 10 min, the supernatants were collected into 1.5 mL centrifuge tubes and stored at −80°C for later use (34, 35). Because of the varying number of groups, for each library, 100 µL of supernatant was pipetted from 5 to 10 individual samples (10–20 µL per sample) and pooled into a new 1.5 mL tube. The newly combined samples were centrifuged at 12,000 × *g* for 5 min at 4°C and filtered through a 0.45 µm filter to remove non-viral particles. RNase and DNase were used to treat the filtrates, followed by digestion of unprotected nucleic acids at 37°C for 60 min (36). Total nucleic acids were then extracted according to the manufacturer’s protocol using the QIAamp MinElute Virus Spin Kit (Qiagen). These nucleic acid samples containing DNA and RNA viral sequences were used for reverse transcription reactions with the SuperScript III reverse transcriptase (Invitrogen) and 100 pmol of a random hexamer primer, followed by a single round of DNA synthesis using Klenow fragment polymerase (New England BioLabs). Libraries were constructed using the Nextera XT DNA Sample Preparation Kit (Illumina) and sequenced on the Illumina NovaSeq platform with 150 bp paired-end reads and dual barcoding.

All steps in the experiment were performed with necessary measures taken to prevent sample cross-contamination and nucleic acid degradation during the process. Aerosol filter tips were used to reduce the probability of sample cross-contamination, and all other experimental materials, including microcentrifuge tubes and tips that came into direct contact with nucleic acid samples, were certified to be free of DNase and RNase. The samples were dissolved in DEPC-treated water containing RNase inhibitors. For blank controls, sterile ddH_2_O was prepared and processed simultaneously under the same experimental conditions. Quality testing was performed using agarose gel electrophoresis and the Agilent bioanalyzer 2100, and no DNA was detected in the control pool. During sequencing on the Illumina NovaSeq platform, the control pool generated a very small number of reads. No viral sequences were found in the control pool when a BLASTx search was performed.

### Assembly of sequence data and identification of HCAPV-1

To reduce host contamination, we acquired the human (*Homo sapiens*) reference genome sequence (GCF_000001405.40) from NCBI and subsequently employed Bowtie2 v2.4.5 (37) to align and remove potential host sequences from the libraries. Primers and low-quality sequences were trimmed using Trim Galore v0.6.5 (https://www.bioinformatics.babraham.ac.uk/projects/trim_galore), with quality control applied using the following options: “--phred33 --length 50 --stringency 3 --paired.” Paired-end reads were assembled using MEGAHIT v1.2.9 (38) with default parameters.

The contigs were aligned against the non-redundant (nr) protein database (downloaded on 2024.05.14) using the BLASTx tool in DIAMOND v2.0.15 (39), with a cut-off E-value of <10^−5^. Viral classification was performed using TaxonKit (40), and the viruses of interest were filtered out from the results. Geneious Prime v2022.0.1 (https://www.geneious.com) was used to predict putative ORFs using default parameters. These predictions were subsequently validated by comparing them to ORFs found in related viruses. Comparisons with the Conserved Domain Database were used for annotating these ORFs. Finally, after filling in the missing regions using Sanger sequencing, we identified a novel parvovirus with a complete genome, designated as HCAPV-1.

### Molecular epidemiology assays

We included the following samples in this epidemiological investigation: (i) 108 lymph node tissues from deceased stray dogs preserved in our laboratory from 2015 to 2024; (ii) whole-body tissues from three other deceased stray dogs collected in 2016, 2018, and 2019 (including submandibular lymph nodes, retropharyngeal lymph nodes, brain, spleen, mesenteric lymph nodes, inguinal lymph nodes, stomach, small intestine, blood, liver, tongue, kidney, lung, heart, gums, and large intestine); (iii) 126 oropharyngeal secretion samples collected in 2024 from pet dogs at veterinary clinics; (iv) 62 oropharyngeal secretion samples from close contacts of pet dogs (including pet owners and veterinarians); and (v) 270 oropharyngeal secretion samples from febrile individuals.

Screening was performed using a nested PCR approach with specifically designed primers. In the first round, all samples were screened with the primers PVP-F1 (5′-ATAGCCTGAAACAAACGGTC-3′) and PVP-R1 (5′-TGTGATGTCTGCTGGTTCTG-3′). This was followed by a second PCR using the primers PVP-F2 (5′-ACCAACTACCATTCACACCC-3′) and PVP-R2 (5′-TCTCCTGTTCTGAGCATTTC-3′), amplifying a 244-nt fragment of the VP gene. The nested PCR procedure involved the following conditions: an initial pre-denaturation at 95°C for 5 min, followed by 31 cycles of denaturation at 95°C for 30 s, annealing at 55°C for 30 s (in both rounds), and extension at 72°C for 30 s, with a final extension step at 72°C for 5 min. The reaction system used the premixed rTaq enzyme (TaKaRa).

### Whole-genome amplification of homologous sequences from HCAPV-1 positive samples

The PCR primers and sequence information used for whole-genome amplification are detailed in Table S1. The reaction conditions were as follows: pre-denaturation at 95°C for 3 min; followed by 35 cycles of denaturation at 95°C for 25 s, annealing at 55°C for 25 s (in both rounds), and extension at 72°C for 5 min.

### Phylogenetic analysis

To elucidate phylogenetic relationships, sequences from different parvovirus groups were downloaded from the GenBank database, along with sequences of proposed species pending ratification. Nucleotide or protein sequences were aligned using MUSCLE in MEGA-11 (41). Sites containing more than 50% gaps were temporarily removed from the alignments. Maximum likelihood trees were then constructed using IQ-TREE v1.6.12 (42). All phylogenetic trees were created using IQ-TREE with 1,000 bootstrap replicates (-bb 1000) and the ModelFinder function (-m MFP). Interactive Tree Of Life was used for visualizing and editing phylogenetic trees (43).

### Prediction of potential genome recombination events

Potential recombination events were analyzed and filtered using the default algorithm of RDP5 software (28), which includes RDP, GENECONV, BootScan, MaxChi, Chimera, SiScan, 3Seq, LARD, and Phylpro. The identified recombination events were further validated using SimPlot software (44).

## Data Availability

The HOS114-116 libraries have been deposited into the National Genomics Data Center (NGDC) of China (https://ngdc.cncb.ac.cn) under the BioProject accession number PRJCA029985. The sequences reported in this paper have been deposited in the GenBase (45) in the National Genomics Data Center (46), Beijing Institute of Genomics, Chinese Academy of Sciences/China National Center for Bioinformation, under accession numbers C_AA085195.1–C_AA085203.1, which are publicly accessible at https://ngdc.cncb.ac.cn/genbase. In addition, the Sanger sequencing data (https://doi.org/10.5281/zenodo.13918682) have been deposited in the Zenodo database.

## SUPPLEMENTAL MATERIAL

Fig. S1 spectrum.03327-25-s0001.pdf Pairwise comparison of the complete genome sequences of HCAPV-1 and its variants.

Fig. S2 spectrum.03327-25-s0002.pdf Maximum likelihood phylogenetic trees of the nucleotide sequences of the whole genome, as well as the NS1 and VP1 genes of HCAPV-1 and its variants.

Fig. S3 spectrum.03327-25-s0003.pdf Distance matrix analysis of HCAPV-1 and its variants.

Fig. S4 spectrum.03327-25-s0004.pdf Secondary structure prediction of HCAPV-1 and its variants.

Table S1 spectrum.03327-25-s0005.docx Primers used for whole genome amplification.

Table S2 spectrum.03327-25-s0006.docx Confirmation of HCAPV-1 recombination detection methods.

ASM does not own the copyrights to Supplemental Material that may be linked to, or accessed through, an article. The authors have granted ASM a non-exclusive, world-wide license to publish the Supplemental Material files. Please contact the corresponding author directly for reuse.
