# Supplementary material for: A novel parvovirus circulating in canine populations and sporadically detected in human oropharyngeal samples
Source: Microbiol Spectr. 2026 Feb 9;14(3):e03327-25. doi: 10.1128/spectrum.03327-25 (PMC12955472; doi:10.1128/spectrum.03327-25)
Supplement: Fig. S1 — Pairwise comparison of the complete genome sequences of HCAPV-1 and its variants. [file spectrum.03327-25-s0001.pdf]

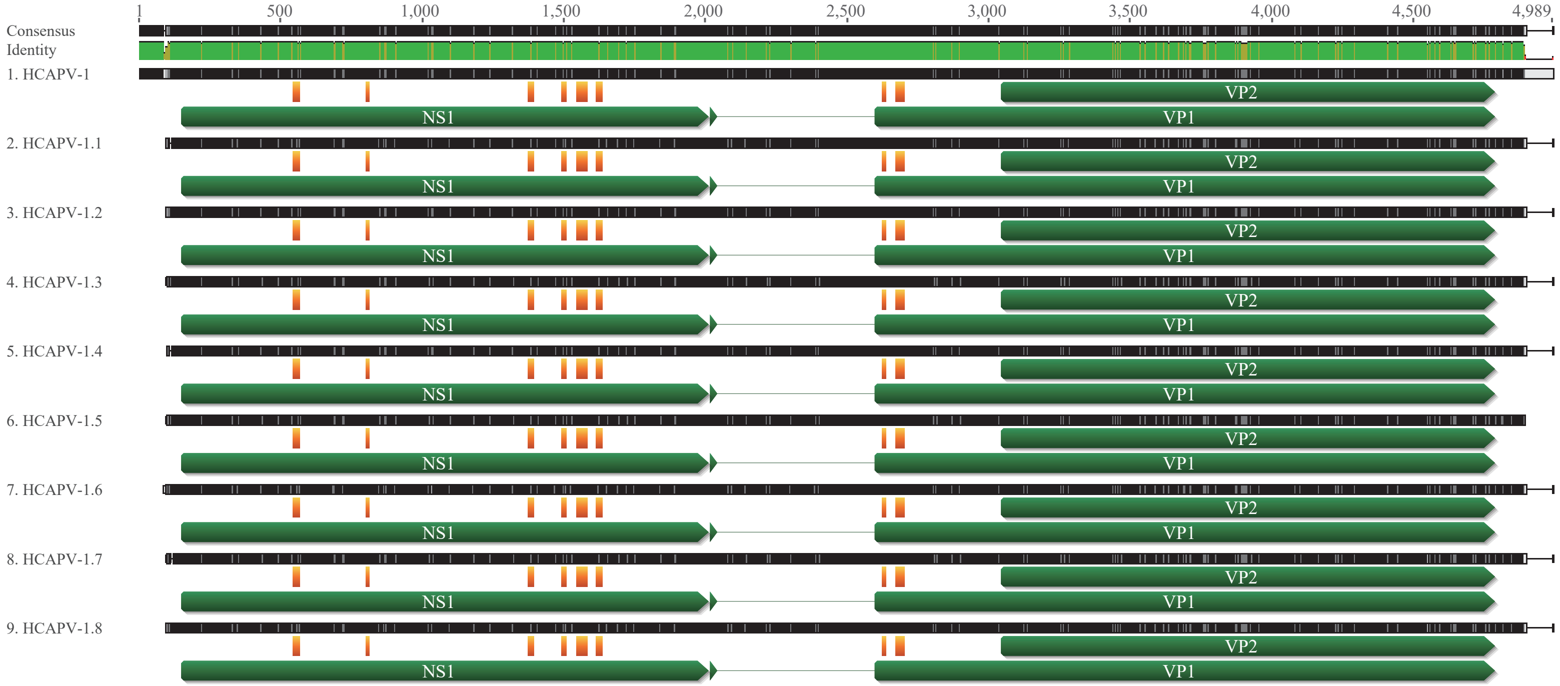

	HCAPV-1	HCAPV-1.1	HCAPV-1.2	HCAPV-1.3	HCAPV-1.4	HCAPV-1.5	HCAPV-1.6	HCAPV-1.7	HCAPV-1.8
HCAPV-1		96.71	96.79	96.59	96.71	98.57	96.64	96.51	96.94
HCAPV-1.1	96.71		99.92	99.77	99.94	99.84	99.79	98.52	98.44
HCAPV-1.2	96.79	99.92		99.79	99.92	99.84	99.79	98.54	98.50
HCAPV-1.3	96.59	99.77	99.79		99.83	99.84	99.73	98.42	98.40
HCAPV-1.4	96.71	99.94	99.92	99.83		99.89	99.83	98.56	98.50
HCAPV-1.5	98.57	99.84	99.84	99.84	99.89		99.80	98.49	98.51
HCAPV-1.6	96.64	99.79	99.79	99.73	99.83	99.80		98.44	98.50
HCAPV-1.7	96.51	98.52	98.54	98.42	98.56	98.49	98.44		98.71
HCAPV-1.8	96.94	98.44	98.50	98.40	98.50	98.51	98.50	98.71	

Extended Data Fig. 1 | A pairwise comparison of the complete genome sequences of HCAPV-1 and its variants. Yellow boxes indicate conserved motifs, as explained in Fig. 1. Darker backgrounds indicate higher sequence identity, while lighter backgrounds indicate lower identity. The table shows the nucleotide identity of the complete genomes of HCAPV-1 and its variants.
