# Supplementary material for: A novel parvovirus circulating in canine populations and sporadically detected in human oropharyngeal samples
Source: Microbiol Spectr. 2026 Feb 9;14(3):e03327-25. doi: 10.1128/spectrum.03327-25 (PMC12955472; doi:10.1128/spectrum.03327-25)
Supplement: Fig. S2 — Maximum likelihood phylogenetic trees of the nucleotide sequences of the whole genome, as well as the NS1 and VP1 genes of HCAPV-1 and its variants. [file spectrum.03327-25-s0002.pdf]

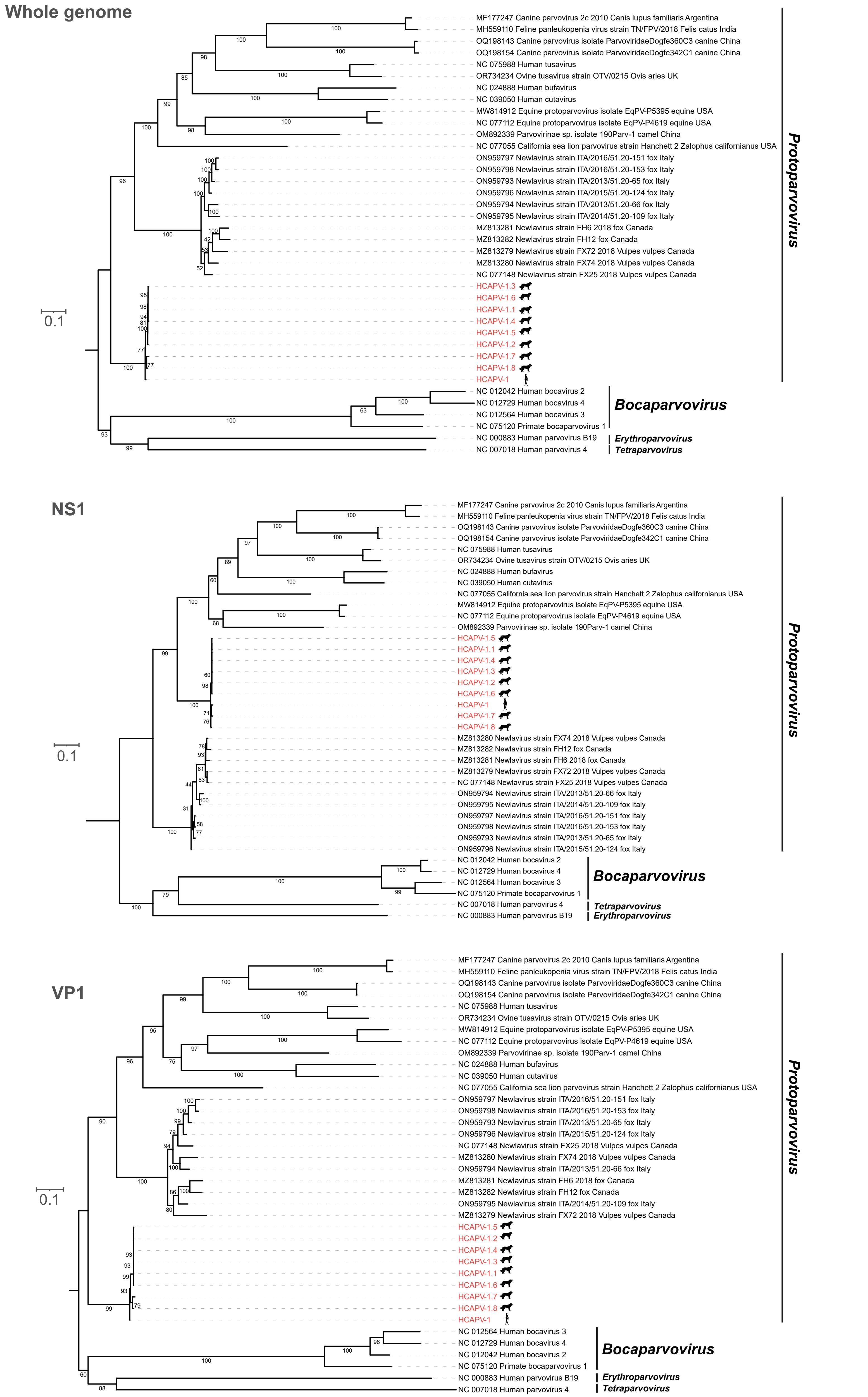

Extended Data Fig. 2 | Maximum likelihood phylogenetic trees of the nucleotide sequences of the whole genome, as well as the NS1 and VP1 genes of HCAPV-1 and its variants. Viruses identified in this study are highlighted in red. Numbers along the branches indicate the percentage of bootstrap values. The scale bar represents the number of substitutions per site. All animal and other life form silhouettes are sourced from PhyloPic (https://www.phylopic.org) and are available for reuse under Creative Commons licenses.
