# Supplementary material for: A novel parvovirus circulating in canine populations and sporadically detected in human oropharyngeal samples
Source: Microbiol Spectr. 2026 Feb 9;14(3):e03327-25. doi: 10.1128/spectrum.03327-25 (PMC12955472; doi:10.1128/spectrum.03327-25)
Supplement: Fig. S3 — Distance matrix analysis of HCAPV-1 and its variants. [file spectrum.03327-25-s0003.pdf]

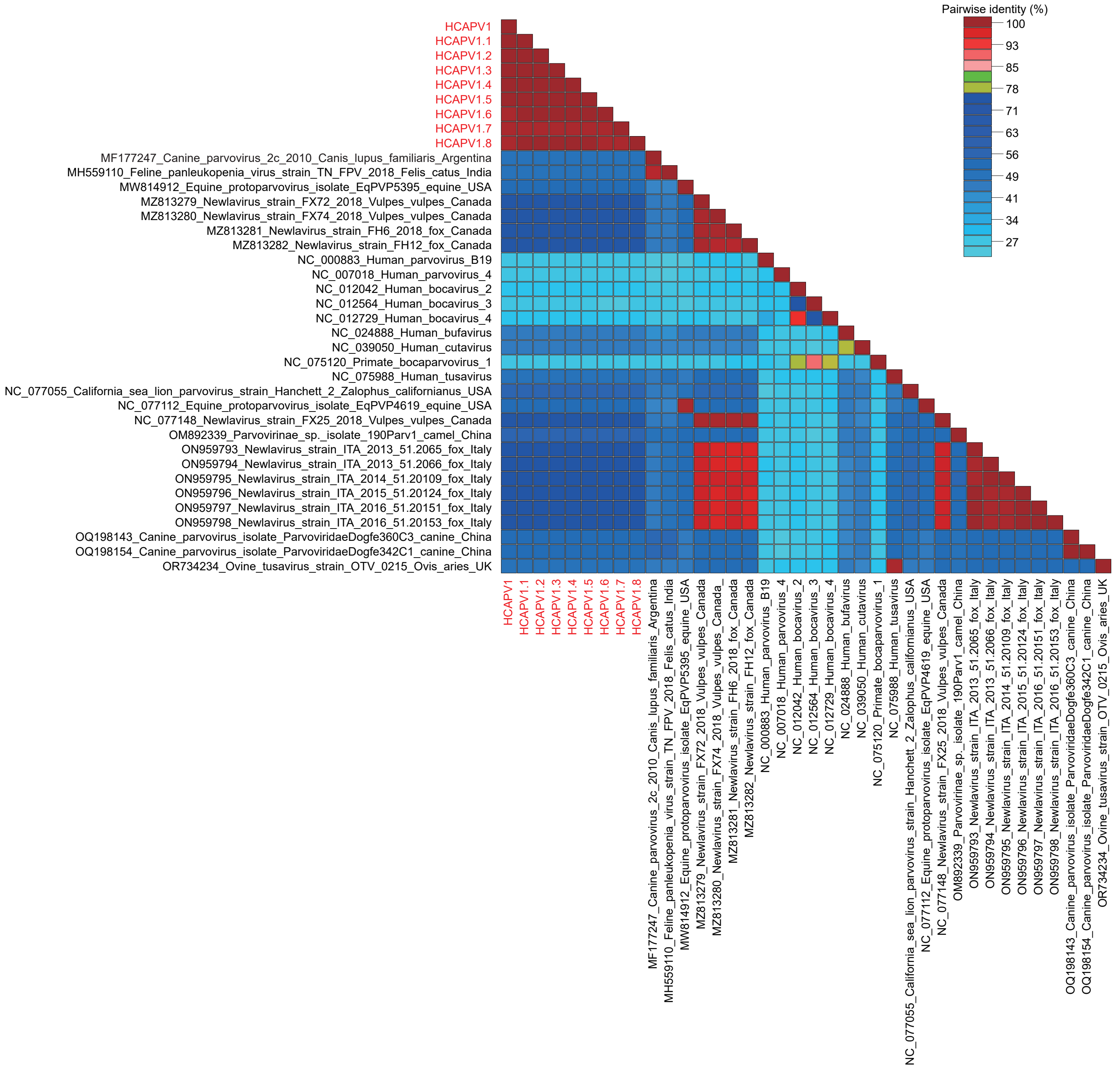

Extended Data Fig. 3 | Distance matrix analysis of HCAPV-1 and its variants. Pairwise sequence comparisons were performed using NS1 protein sequences within a maximum likelihood consensus tree.
