# Supplementary material for: A novel parvovirus circulating in canine populations and sporadically detected in human oropharyngeal samples
Source: Microbiol Spectr. 2026 Feb 9;14(3):e03327-25. doi: 10.1128/spectrum.03327-25 (PMC12955472; doi:10.1128/spectrum.03327-25)
Supplement: Fig. S4 — Secondary structure prediction of HCAPV-1 and its variants. [file spectrum.03327-25-s0004.pdf]

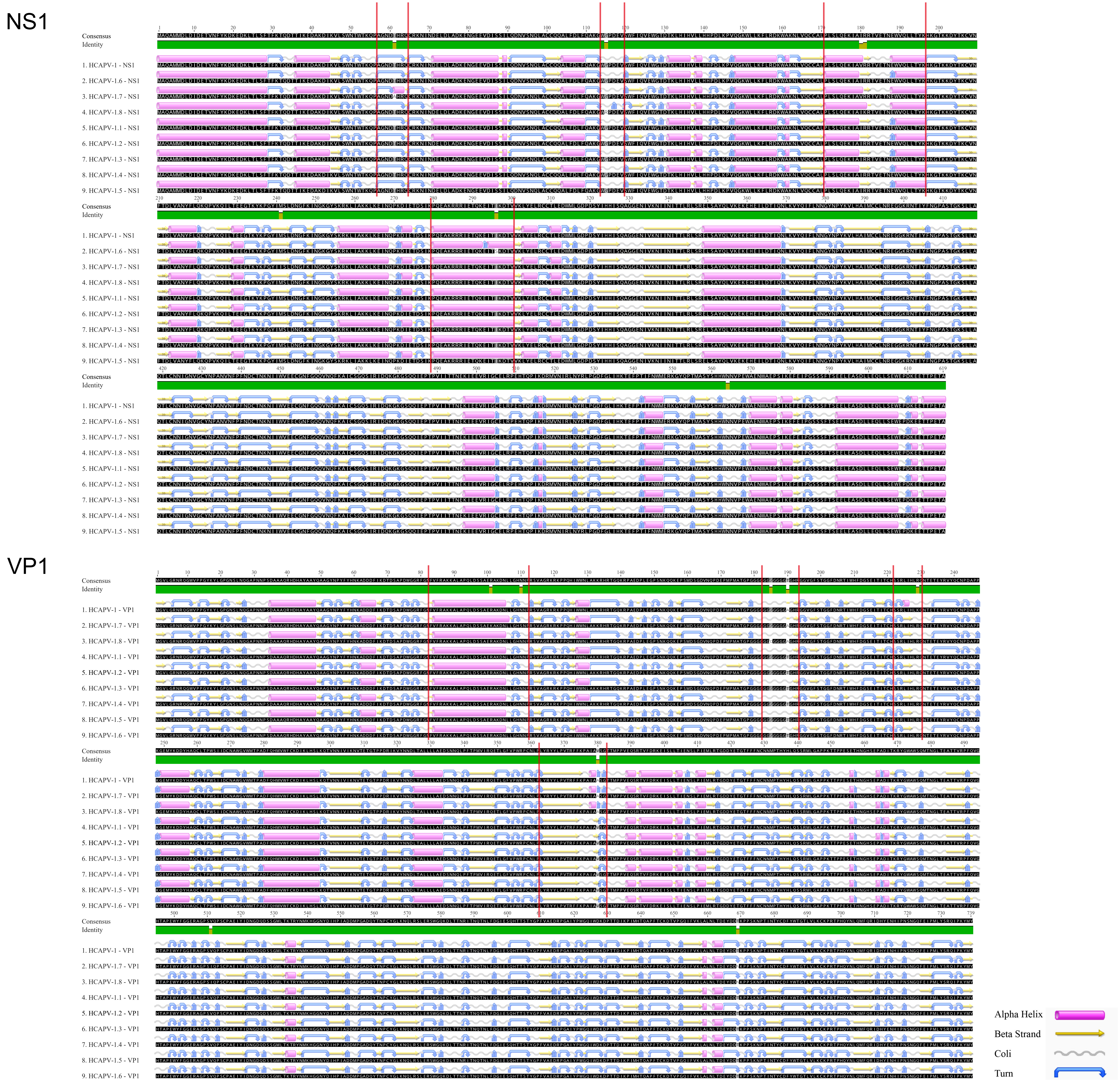

Extended Data Fig. 4 | Secondary structure prediction of HCAPV-1 and its variants. The secondary structure of the NS1 and VP1 proteins of HCAPV-1 and its variants was predicted using the EMBOSS tool Garnier. Predicted alterations in the secondary structure are marked by red vertical lines.
