# Supplementary material for: A novel parvovirus circulating in canine populations and sporadically detected in human oropharyngeal samples
Source: Microbiol Spectr. 2026 Feb 9;14(3):e03327-25. doi: 10.1128/spectrum.03327-25 (PMC12955472; doi:10.1128/spectrum.03327-25)
Supplement: Table S1 — Primers used for whole genome amplification. [file spectrum.03327-25-s0005.docx]

| **Supplementary Table 1 \| Primers used for whole genome amplification** | | | | |
| --- | --- | --- | --- | --- |
| **Primer** | **Targeted region** | **Sequence (5**'**-3**'**)** | **Fragment size (bp)** | **Annealing temperature (^o^C)** |
| P1-L1 (o) | 24-1,139 | GAGCGAAGACGATTGGTTAG | 1,116 | 55 |
| P1-R1 (o) |  | CCTGCTTGAGAGATGTGATG |  |  |
| P1-L2 (i) | 64-1,114 | AGTGATTGGTTGGACAGAGG | 1,051 | 55 |
| P1-R2 (i) |  | AGGAGTCTGGGTCTCCTAGC |  |  |
| P2-L1 (o) | 975-2,160 | TGACAGCATAAAACCACAGG | 1,186 | 55 |
| P2-R1 (o) |  | ATTCCAGCACATATTCATCG |  |  |
| P2-L2 (i) | 996-2,089 | AGCTAAACGCAGAAGAATCG | 1,094 | 55 |
| P2-R2 (i) |  | TGAATATAAGCCAGCCAAGG |  |  |
| P3-L1 (o) | 1,926-3,164 | AGAACTCGAAGCATCTGACC | 1,239 | 55 |
| P3-R1 (o) |  | CTAAAACCAACACCACCACG |  |  |
| P3-L2 (i) | 1,957-3,089 | CTTCTCTCCGAGTGGGAACC | 1,133 | 55 |
| P3-R2 (i) |  | ATTGGCATGGGTTCGTCTGG |  |  |
| P4-L1 (o) | 2,924-4,116 | AACGAAAACCACCTCAACAC | 1,193 | 55 |
| P4-R1 (o) |  | AGGGCAAGATGGTTGTACAG |  |  |
| P4-L2 (i) | 2,986-4,086 | AAAAGACCAGCAGAAGACCC | 1,101 | 55 |
| P4-R2 (i) |  | TCTTTCCCCTCCAAAGTACC |  |  |
| P5-L1 (o) | 3,914-4,948 | AAACAACACCGCCAGAATCC | 1,035 | 55 |
| P5-R1 (o) |  | ATGGAAAAGTGCATAGACCC |  |  |
| P5-L2 (i) | 3,945-4,924 | TGGACATTCAGAACCAGCAG | 980 | 55 |
| P5-R2 (i) |  | AACCCACCCTAACTAAACGC |  |  |
| The letter 'o' represents outer primers, and the letter 'i' represents inner primers. | | | | |
