# Supplementary material for: A novel parvovirus circulating in canine populations and sporadically detected in human oropharyngeal samples
Source: Microbiol Spectr. 2026 Feb 9;14(3):e03327-25. doi: 10.1128/spectrum.03327-25 (PMC12955472; doi:10.1128/spectrum.03327-25)
Supplement: Table S2 — Confirmation of HCAPV-1 recombination detection methods. [file spectrum.03327-25-s0006.docx]

| **Supplementary Table 2 \| Confirmation table for HCAPV-1 recombination detection methods** | | |
| --- | --- | --- |
| Methods | # seqs detected in | Av. P-Val |
| RDP | 1 | 5.549×10^-04^ |
| GENECONV | - | - |
| BootScan | - | - |
| MaxChi | - | - |
| Chimaera | - | - |
| SiScan | 1 | 1.309×10^-13^ |
| 3Seq | 1 | 2.836×10^-02^ |
| LARD | - | - |
| Phylpro | - | - |
